# The Neutrophil-to-Monocyte Ratio and Platelet-to-White Blood Cell Ratio Represent Novel Prognostic Markers in Patients with Pancreatic Cancer

**DOI:** 10.1155/2021/6693028

**Published:** 2021-05-24

**Authors:** Feng Tang, Penghui Dai, Qiongqiong Wei, Ke Gan, Zijie Wang, Huan Chen, Ting Li, Muhan Lv, Mingming Deng, Gang Luo

**Affiliations:** Department of Gastroenterology, The Affiliated Hospital of Southwest Medical University, Luzhou, Sichuan 646000, China

## Abstract

**Background:**

Inflammation plays an important role in the development of tumors. Several serum based-markers and ratios have been investigated for their prognostic value in pancreatic cancer. However, the prognostic value of the neutrophil-to-monocyte ratio (NMR) and platelet-to-white blood cell ratio (PWR) for patients with pancreatic cancer has scarcely been investigated.

**Methods:**

From October 2013 to November 2018, a retrospective cohort study was performed on 269 pancreatic cancer patients without treatment. Receiver operating characteristic curves were generated, and areas under the curve were compared for the evaluation of the discriminatory ability of inflammation-based prognostic scoring systems. Kaplan-Meier curves and the Cox proportional hazard model were employed to analyze the relationships among NMR, PWR, and overall survival (OS).

**Results:**

The optimal cutoff values of NMR and PWR were 48 and 6, respectively. In univariate analysis, the survival time of NMR > 48 and PWR ≤ 6 was shorter than that of NMR ≤ 48 and PWR > 6 in patients with pancreatic cancer (*P* < 0.001). In Cox univariate and multivariate analyses, NMR (hazard ratio (HR), 9.095; 95% confidence interval (CI), 3.64–22.72; *P* < 0.001) and PWR (HR, 8.230; 95% CI, 3.32–20.43; *P* < 0.001) were significantly correlated with OS.

**Conclusions:**

The current study demonstrated that NMR and PWR may serve as novel and promising inflammatory prognostic scores for patients with pancreatic cancer. Elevated NMR (>48) and depressed PWR (<6) were independently associated with poor prognosis in patients with pancreatic cancer.

## 1. Introduction

Pancreatic cancer, known as the “king of cancer,” is one of the worst prognostic malignancies. Its 1- and 5-year survival rates are 21% and 3%, respectively [1]. In 2018, the top three regions for the incidence and mortality of pancreatic cancer worldwide were Asia, Europe, and North America [2]. Pancreatic cancer is expected to increase its rank from the third to the second leading cause of cancer deaths in the United States in 2030 [3]. In 2015, the incidence of pancreatic cancer in China is approximately 90,100, and the annual death toll is approximately 79,400 and is increasing annually [4]. The histopathology and genetic mutations of pancreatic cancer are complex, and it is difficult to accurately predict the invasion of the disease [5]. More than 80% of patients have been diagnosed with stage III or IV, with limited surgical opportunities. However, the survival times of advanced patients vary considerably [6]. To date, no breakthrough has been achieved in the treatment of pancreatic cancer, and no widespread and effective prognostic indicators for patients with pancreatic cancer are available [7], so more in-depth researches should be conducted on pancreatic cancer.

In 1863, Virchow first discovered the correlation between inflammation and malignant tumors [8]. Cancer-associated inflammation is currently recognized as the seventh marker of cancers [9]. Systemic inflammatory response is an independent influencing factor for the prognosis of many malignant tumors and plays an important role in promoting the occurrence, development, and metastasis of tumors [10]. Activation of oncogenes or inactivation of tumor suppressor genes can lead to transcription of inflammatory mediators, producing tumor-associated inflammatory microenvironment in the body [11]. The activation of the WNT and WNT2 pathway rendered a peculiar immune microenvironment in the lymph node positive for pancreatic ductal adenocarcinoma that promotes invasiveness, epithelial mesenchymal transition, and metastatic potential [12]. Myeloid-derived suppressor cells (MDSCs), one of the major cell populations comprised of macrophages, dendritic cells (DCs), and granulocytes, strongly expand in pathological situations such as cancer. High levels of circulating MDSCs are detected in patients with pancreatic cancer and associated with the poor OS of patients [13]. Circulating white blood cells and acute-phase proteins that respond to advanced cancer were massively activated in the clinical laboratory measures [14]. Existing researches show the significance of the peripheral blood cell count and ratios in predicting the survival time in a variety of tumors, such as kidney cancer [15], colon cancer [16], non-small-cell lung cancer [17], and pancreatic cancer [18]. As for patients with pancreatic cancer, a low neutrophil-to-lymphocyte ratio (NLR) and monocyte-to-lymphocyte ratio (MLR) are significantly associated with improved overall survival (OS) [19], and patients with elevated platelet-to-albumin ratios (PAR) have poor median disease-free-survival and OS [20]. The elevated platelet-to-lymphocyte ratio (PLR) is associated with poor prognosis of pancreatic cancer [21]. The neutrophil-to-albumin ratio combined with CA199 (NARCA) can only be used only for CA199-positive patients and has limitations [22]. However, the conclusions in these studies remain controversial. Thus, the more sensitive markers are needed to improve prognosis. We designed a retrospective cohort study to compare and evaluate the prognostic significance of different inflammatory markers in patients with pancreatic cancer.

## 2. Materials and Methods

### 2.1. Subjects

**Figure 1:**
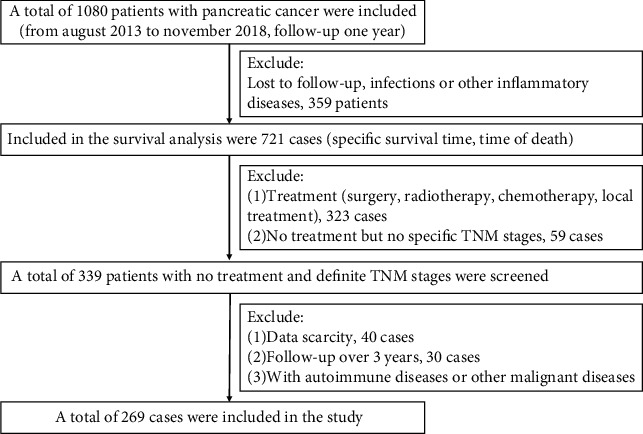
Research route.

The medical records of a total of 1080 patients diagnosed with pancreatic cancer in the Affiliated Hospital of Southwest Medical University from August 2013 to November 2018 were carefully reviewed (Figure 1). Only patients with the pathological diagnosis of pancreatic ductal adenocarcinoma were included. The exclusive criteria were as follows: lost to follow-up, patients lacking routine blood indexes or imaging data, patients with clinical evidence of infection or other inflammatory diseases, treatment (surgery, chemotherapy, radiotherapy, and local treatment), and complication with autoimmune diseases and other malignancy. Finally, 269 patients who were untreated and pathologically diagnosed with pancreatic cancer were included in the retrospective cohort study.

Clinicopathological variables including age, gender, CA199, TNM stage, and survival outcomes were collected. The influence of treatment on survival time was prevented by collecting blood routine indexes in the first peripheral blood sample, including white blood cell count, neutrophil count, monocyte count, lymphocyte count, eosinophil count, basophil count, platelet count, and albumin level.

The inflammation-based prognostic scores in this study were defined as follows: lymphocyte-to-monocyte ratio (LMR), platelet-to-lymphocyte ratio (PLR), neutrophil-to-lymphocyte ratio (NLR), absolute count of neutrophils divided by the absolute white cell count minus the absolute count of neutrophils (dNLR), neutrophil-to-monocyte ratio (NMR), platelet-to-white blood cell ratio (PWR), platelet-to-neutrophil ratio (PNR), platelet-to-monocyte (PMR), and platelet-to-albumin ratio (PAR). The study was approved by the Ethics Board of the Affiliated Hospital of Southwest Medical University.

### 2.2. Follow-Up

All patients were followed-up for one year through telephone interviews. OS was defined as the time from the date of initial diagnosis to the date of death from any cause.

### 2.3. Statistical Analysis

EpiData was used for data entry in this study. Continuous variable was described using mean ± standard deviation or *Q* (P25-P75) and was compared using *t*-test or rank sum test. Categorical variables are presented as number of patients (%), and the methods of the *χ*^2^ test were used for statistical inference. For the evaluation of the discriminatory ability of the inflammation-based prognostic scoring systems, receiver operating characteristic curves (ROC) were generated, and differences among the areas under the curve (AUC) were compared. And sensitivity, specificity, and Youden index (YI) were used in identifying optimal cutoff values. Survival time was described statistically using the median survival time and its 95% confidence interval (CI). Comparisons between groups were statistically inferred using the log-rank test. Univariate and multivariate analyses of factors considered prognostic of OS were performed using the Cox proportional hazards model. Hazard ratios (HRs) and corresponding 95% CIs of all variables were calculated. All statistical analyses were performed using SPSS software version 25.0. A two-sided *P* value of <0.05 was considered statistically significant.

## 3. Results

A total of 269 patients were included in the retrospective cohort study. The clinicopathological characteristics of patients are presented in Table 1. The mean age of the study cohort was 67.98 years (range 41-91). Of all the patients, 155 (57.6%) patients were male and 114 (42.4%) patients were female. 105 (39.0%) patients are <65 years, and 164 (61.0%) patients are ≥65 years. 45 patients (16.7%) are in stages I-III, and 224 patients (83.3%) are in stage IV. After 1-year follow-up, 260 patients died (96.7%) and 9 patients (3.3%) survived. The median survival time was 2.0 months (1.0-4.0 months).

Patients were stratified into two cohorts by median survival time (≤2 months and >2 months, respectively). Correlation between the survival time and the clinicopathological characteristics was analyzed (Table 2). Survival time was associated with CA199 (*P* = 0.002, AUC =0.606), white blood cell count (*P* = 0.037, AUC =0.574), neutrophil count (*P* = 0.011, AUC =0.590), lymphocyte count (*P* = 0.018, AUC =0.583), NMR (*P* = 0.001, AUC =0.992), PWR (*P* = 0.001, AUC =0.992), and other inflammatory indicators (LMR, PLR, NLR, and dNLR, all *P* < 0.05).

### 3.1. Cutoff Values, Sensitivity, and Specificity for NMR and PWR

Both NMR and PWR had the largest AUC values (0.992) which were chosen to be candidate markers (Table 2). Optimal cutoff values were determined by sensitivity, specificity, and YI. As shown in Table 3, when the cutoff value of NMR was 48, the sensitivity, specificity, and YI were 1, 0.984, and 0.984, respectively. And when the cutoff value of PWR was 6, the sensitivity, specificity, and YI were 1, 0.992, and 0.992, respectively (Table 4).

### 3.2. Association between Median Survival Time and Clinicopathological Characteristics

**Figure 2:**
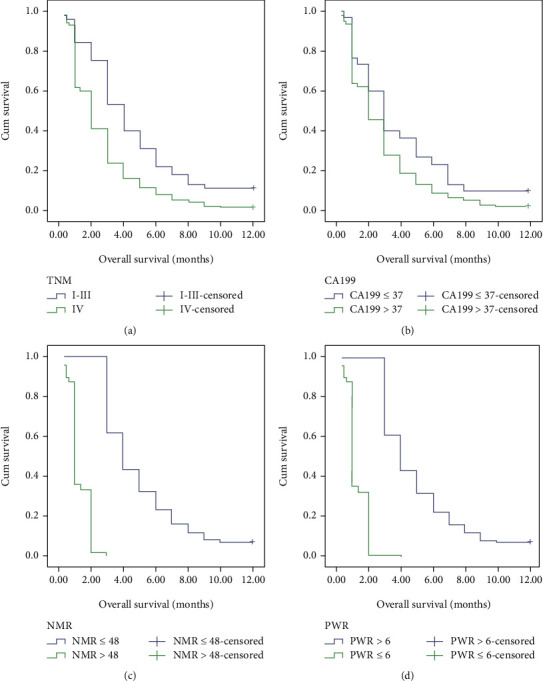
Kaplan-Meier curves for cumulative survival. (a) The OS of TNM I-III stages was longer than that of stages IV (*P* < 0.001). (b) The OS of patients whose CA199 < 37 was longer than CA199 ≥ 37 (*P* = 0.034). (c) The OS of patients with pancreatic cancer with NMR ≤ 48 is longer than that of patients with NMR > 48 (*P* < 0.001). (d) The OS of patients with pancreatic cancer with PWR > 6 is longer than that of patients with PWR ≤ 6 (*P* < 0.001).

The cutoff values of NMR and PWR were 48 and 6, respectively, for the prediction of prognosis in patients with pancreatic cancer. In univariate analysis (Table 5 and Figure 2), the median survival time (MST) has no significant difference in age and gender (*P* = 0.320 and *P* = 0.765, respectively). In accordance with previous studies, the TNM stage (stages I-III vs. stage IV) and CA199 (<37 vs ≥37) were significantly associated with MST (*P* < 0.001 and *P* < 0.034, respectively). In addition, patients with NMR ≤ 48 had longer MST than patients with NMR > 48 (*P* < 0.001), and patients with PWR > 6 had longer MST than patients with PWR ≤ 6 (*P* < 0.001).

### 3.3. Univariate and Multivariate Cox Analyses for OS

Next, we validate the results in the Cox regression model. In univariate Cox analysis (Table 6), OS was related to the TNM stage (HR, 1.80; 95% CI 1.28-2.53; *P* < 0.001), CA199 (HR, 1.449; 95% CI 0.97-2.17; *P* = 0.072), NMR (HR, 19.994; 95% CI 12.62-31.68; *P* < 0.001), and PWR (HR, 19.592; 95% CI 12.49-30.74; *P* < 0.001). In multivariate Cox regression (Table 7), NMR and PWR still showed significant survival predictive value after being adjusted for the factors described above. Patients with NMR > 48 had poorer OS than patients with NMR ≤ 48 (HR, 9.095; 95% CI 3.64-22.72; *P* < 0.001). Patients with PWR > 6 had better OS than patients with PWR ≤ 6 (HR, 8.23; 95% CI 3.32-20.43; *P* < 0.001).

## 4. Discussion

Our study indicated that NMR and PWR may serve as the independent prognostic markers in patients with pancreatic cancer. To the best of our knowledge, this was the first study that investigated the prognostic value of NMR and PWR in pancreatic cancer. First, our study found that the white blood cell count, neutrophil count, lymphocytes, and most blood cell ratios were statistically significant (Table 2; *P* < 0.05). Second, the blood cell ratio, such as NMR, PWR, NLR, LMR, and PLR had better predictive value than leukocytes, lymphocytes, and neutrophils alone. The platelet count, monocyte count, PMR, and PNR have no predictive significance for pancreatic cancer. Moreover, the present study demonstrates that NMR and PWR have high accuracy in predicting the prognosis of patients with pancreatic cancer, with an AUC of 0.992 for NMR and PWR. In univariate Cox analysis, patients with NMR > 48 died at 19.994 times the rate of those with NMR ≤ 48 (HR, 19.994; 95% CI: 12.62-31.68; *P* < 0.001) during follow-up, and those with PWR ≤ 6 at 19.592 times the rate of those with PWR > 6 (HR, 19.592; 95% CI: 12.484-30.743; *P* < 0.001). Interestingly, patients in stage IV died only at 1.80 times the rate of patients in stages I-III (HR, 1.80; 95% CI: 1.28-2.53; *P* < 0.001) in univariate analysis. And in multivariate Cox analysis, patients with NMR > 48 had a 9.095-fold higher risk of death than those with NMR ≤ 48 (HR, 9.095; 95% CI 3.64-22.72; *P* < 0.001) and patients with PWR ≤ 6 had an 8.23-fold higher risk of death than those with PWR > 6 (HR, 8.23; 95% CI 3.32-20.43; *P* < 0.001). Therefore, we speculate that NMR and PWR have strong distinguishing ability and are potential prognostic markers for OS in pancreatic cancer. Our results indicated that the elevated NMR and the decreased PWR had poor prognosis for patients with untreated pancreatic cancer. Most importantly, all easily assessed and predominantly widely used variables were integrated, concluding that NMR and PWR were more representative and reflective predictors for pancreatic cancer. They may reflect the systemic response of the host to the tumors.

Systemic inflammatory response played an important role in tumor growth and metastasis. Changes in blood cell counts were associated with clinical outcomes of various tumors. The results of multiple previous studies on resected pancreatic cancer are inconsistent and convoluted by some common problems, such as the small number of patients, unclear selection of the optimal cutoff values [23, 24], and concomitant evaluation of resectable and unresectable disease [25]. Moreover, most studies were aimed at patients with resected pancreatic cancer. By contrast, the patients included in the present study were all untreated, and thus, the progress of the disease in a natural state was effectively monitored, and the survival times of patients were reliably predicted.

Our study suggests that the elevated NMR is independently associated with poor prognosis in patients with pancreatic cancer. Neutrophils can secrete important cytokines and chemokines, such as vascular endothelial growth factor and matrix metalloproteinase, promote angiogenesis in the tumor microenvironment, and support tumor growth and metastasis; in addition, it can suppress the immune activity of lymphocytes and natural killer cells to facilitate tumor growth [26, 27]. As is well known, tumors grow and evolve through constant crosstalk with surrounding microenvironment, and angiogenesis and immunosuppression frequently occur in response to this crosstalk [28]. What is more, the elevated neutrophil counts might reflect tumor progression by providing an appropriate environment for tumor growth. For example, in patients with renal cell carcinoma, an increased neutrophil count was significantly associated with tumor size and a decreased survival time [29]. In a study cohort of 1,410 patients with nasopharyngeal carcinoma, elevated neutrophil counts led to poor OS [30]. Although the specific mechanism was unclear, there were multifactorial effects on neutropenia [31]. The elevated neutrophil count was related to the increased composition of immature cells and might change the functional status of the body. These changes would create an immunosuppressive environment, thereby weakening the function of immune cells and promoting the aggressive growth of tumors [32]. Monocytes were the third largest component of leukocytes, and increased monocyte counts indicated poor survival of solid tumors [33]. However, in the present study, monocyte count was within the normal range, and there was no correlation between the monocyte count and the MST of pancreatic cancer. The lymphocyte count was significantly correlated with the survival time of patients, consistent with that in the previous study. The lower lymphocyte count decreased the immune response to tumor and leads to proliferation and metastasis of the tumor. PD-1 is an important molecule for T cell suppression and might affect the cytotoxic capability of T lymphocytes in tumor microenvironment [34]. In patients with pancreatic cancer, high PD-1 expression level on CD8+T lymphocytes is associated with the poor OS [35]. Meanwhile, the increased neutrophil count may suppress the cytotoxic activity and count of lymphocytes and contribute to poor survival time. Therefore, the prediction of survival time for pancreatic cancer with NMR was mainly based on the changes of neutrophils. NMR was elevated because of the increase in neutrophil count, and elevated NMR was associated with short survival time. Similarly, our study showed that the elevated PWR was associated with longer survival times. Previous studies had shown that thrombocytopenia hinders tumor metastasis [36], and increased platelet count promoted metastasis and might be associated with poor OS of patients with pancreatic cancer [37]. However, in our study, platelet levels were within the normal range, and the median survival time of patients with pancreatic cancer are not statistically related to platelets in univariate analysis.

Therefore, change in PWR mainly contributes to the increased white blood cell count. The number and subsets of the white blood cell have been analyzed in cancer patients as predictive biomarkers for several decades [28]. Changes in the white blood cell count affect the clinical outcome of various cancers, such as myeloma and colorectal cancer, and increase in the white blood cell count is associated with shorter survival time [38, 39]. Similarly, the increase in white blood cell count had a poorer prognosis in breast cancer [40] and gastric cancer [41]. And neutrophils were the main components of white blood cells, so it could be reasonably speculated that the increase in white blood cells was mainly due to the increase in neutrophils. Increased NMR and decreased PWR were negatively correlated with OS. In Cox multivariate analysis, NMR and PWR were the independent predictors of pancreatic cancer.

Two previous studies on 381 and 442 patients with resectable pancreas indicated that NLR < 2 (HR, 1.51, 95% CI 1.15–1.99; *P* < 0.003) [42] and NLR < 5 (HR, 1.66; 95% CI 1.12–2.46; *P* < 0.012) [43] were independent predictors of pancreatic cancer. A study in a group of 144 curatively resected pancreatic cancers showed that LMR ≥ 2.86 was an independent favorable prognostic factor with HR 0.15 (95% CI 0.085–0.252*P* < 0.001) [44]. A similar observation was reported in another resectable and advanced cancer study showing that LMR > 2.8 (HR = 0.81, 95% CI 0.66-0.99*P* < 0.040) reduced the risk of death [45]. In addition, controversy existed as to whether PLR was an independent predictor. PLR with the optimal cutoff value of 150 in patients with resectable pancreatic cancer was associated with survival time [46]. However, the prognostic implications of PLR with the same cutoff value were not observed in unresectable cases [47].

TNM stages and CA199, as promising prognostic makers for pancreatic cancer in many studies, were also investigated in our study. Consistently, TNM stages had predictive significance in Cox univariate analysis (HR, 1.80; 95% CI 1.28-2.53; *P* < 0.001) but had no predicted significance in Cox multivariate analysis. The possible reason was that most of the subjects in our study were stage IV patients (83.3%). CA199 was associated with OS in our study, although the association was not statistically significant (HR,1.449; 95% CI 0.97~2.17;*P* = 0.072). In fact, CA199 as a prognostic marker of pancreatic cancer has also been controversial. Tingle et al. found that CA199 was an independent predictor of survival time in unresectable pancreatic cancer of 145 patients [22]. A study of 307 patients who underwent surgical resection after neoadjuvant chemoradiotherapy showed that CA199 was not associated with disease-specific survival time in patients with locally advanced pancreatic cancer [48]. The prognostic role of CA199 in untreated patients was not significant and needs to be further verified in a larger perspective study. In summary, our study found that elevated NMR (HR, 9.10; 95% CI 3.64-22.72; *P* < 0.001) and depressed PWR (HR,8.23; 95% CI 3.32-20.43;*P* < 0.001) were associated with impaired long-term survival.

This study has some limitations. First, our research is a single center with a relatively small sample size, which might have affected the results. Secondly, there existed a possibility of selection bias, since most of subjects in this study were diagnosed in stage IV (83.3%). This bias might result in reducing the effect of the TNM stage on prognostic analysis. Thirdly, we did not assess the functional status of blood cells. The immune status of the cells might reduce the defense ability of the tumor. Finally, due to the retrospective nature, our study is hypothesis-generating rather than conclusion-forming, and the findings should be interpreted cautiously and validated in prospective studies. Therefore, a multicenter, large-sample, and carefully designed prospective study is warranted in the future.

## Figures and Tables

**Table 1:** Clinicopathologic characteristics.

Variables	*N* (%)	Mean ± SD/median (Q1–Q3)
Age (years)		67.98 ± 11.42
<65	105 (39.0%)	
≥65	164 (61.0%)	
Gender		
Male	155 (57.6%)	
Female	114 (42.4%)	
TNM stages		
I-III	45 (16.7%)	
IV	224 (83.3%)	
Median survival time (months)		2.0 (1.0~4.0)
Survival outcomes		
Death	260 (96.7%)	
Survival	9 (3.3%)	

**Table 2:** Comparison of different blood cell count and inflammatory markers in patients with different survival time groups.

Variables	Survival time > 2 months	Survival time ≤ 2 months	*Z*	*P*	AUC
CA199 (U/mL)	203.4 (30.70-400.00)	400.0 (48.2-400.0)	3.059	0.002	0.606
WBC (×10^9^/L)	6.4 (5.10-8.70)	7.5 (5.3-10.3)	2.089	0.037	0.574
NEU (×10^9^/L)	4.6 (3.50-6.50)	5.7 (3.8-8.0)	2.558	0.011	0.590
Mono (×10^9^/L)	0.43 (0.33-0.62)	0.48 (0.33-0.66)	1.398	0.162	0.594
LYM (×10^9^/L)	1.1 (0.8-1.4)	0.9 (0.7-1.3)	2.361	0.018	0.583
PLT (×10^9^/L)	191.5 (137.0-242.0)	202.0 (147.5-246.5)	1.125	0.260	0.540
BASO (×10^9^/L)	0.03 (0.01-0.06)	0.02 (0.01-0.06)	0.314	0.753	0.511
EOS (×10^9^/L)	0.08 (0.01-0.23)	0.05 (0.01-0.12)	2.645	0.008	0.593
ALB (g/L)	40.4 (37.4-43.2)	37.3 (34.1-42.1)	3.527	<0.001	0.625
LMR	2.6 (1.7-3.6)	2.1 (1.3-3.1)	2.981	0.003	0.605
PLR	169.3 (125.8-233.3)	204.3 (129.1-307.3)	2.860	0.004	0.601
NLR	4.4 (2.8-7.3)	6.0 (3.5-10.0)	3.248	0.001	0.615
dNLR	2.8 (1.8-3.9)	3.2 (2.4-4.9)	2.602	0.009	0.592
NMR	10.9 (7.9-13.2)	115.0 (96.5-130.0)	13.916	<0.001	0.992
PWR	27.8 (20.5-38.3)	4.1 (3.5-4.5)	13.926	<0.001	0.992
PNR	40.9 (25.6-57.4)	36.1 (25.7-50.1)	1.467	0.142	0.552
PMR	403.1 (321.4-595.1)	432.7 (309.4-632.2)	0.018	0.986	0.501
PAR	4.8 (3.5-6.1)	5.2 (3.8-6.8)	2.118	0.034	0.575

WBC: white blood cell; NEU: neutrophil; mono: monocyte; LYM: lymphocyte; PLT: platelet; BASO: basophil; EOS: eosinophil; ALB: albumin; LMR: lymphocyte-to-monocyte ratio; PLR: platelet-to-lymphocyte ratio; NLR: neutrophil-to-lymphocyte ratio; dNLR: absolute count of neutrophils divided by the absolute white cell count minus the absolute count of neutrophils; NMR: neutrophil-to-monocyte ratio; PWR: platelet-to-white blood cell ratio; PNR: platelet-to-neutrophil ratio; PMR: platelet-to-monocyte ratio; PAR: platelet-to-albumin ratio.

**Table 3:** Results of diagnostic indicators at different cutoff values of NMR.

NMR	Sensitivity	Specificity	YI (Youden index)
10	1.000	0.421	0.421
30	1.000	0.960	0.960
48	1.000	0.984	0.984
60	0.958	0.984	0.942
80	0.902	0.992	0.894
100	0.657	0.992	0.649

**Table 4:** Results of diagnostic indicators at different cutoff values of PWR.

PWR	Sensitivity	Specificity	YI (Youden index)
4.0	0.462	0.992	0.454
5.0	0.923	0.992	0.915
6.0	1.000	0.992	0.992
8.0	1.000	0.976	0.976
11.0	1.000	0.913	0.913
12.0	1.000	0.897	0.897

**Table 5:** Comparison of median survival time and 95% CI at different levels of variables.

Variables	Median survival time and 95% CI (month)		*N*	*χ* ^2^	*P*
	Low-level group (0 or 1)	High-level group (1 or 2)			
Age (<65/≥65)	2.0 (1.5-2.5)	2.0 (1.7-2.3)	105/164	0.988	0.320
Gender (M/F)	2.0 (1.6-2.4)	2.0 (1.6-2.4)	155/114	0.089	0.765
TNM stages	4.0 (3.2-4.8)	2.0 (1.7-2.2)	45/224	16.435	<0.001
CA199	3.0 (2.1-3.9)	2.0 (1.7-2.3)	30/204	4.510	0.034
NMR	4.0 (3.5-4.5)	1.0 (0.95-1.05)	124/145	266.357	<0.001
PWR	4.0 (3.5~4.5)	1.0 (0.95-1.05)	125/144	265.941	<0.001

**Table 6:** Univariate Cox analysis at different variables for OS.

Variables	*β*	Wald	*P*	Hazard ratio (95% CI)
Age (years)	0.108	0.716	0.398	1.11 (0.87-1.43)
Gender	0.032	0.065	0.799	1.03 (0.81-1.32)
TNM stages	0.589	0.174	<0.001	1.80 (1.28-2.53)
CA199	0.371	3.244	0.072	1.449 (0.97-2.17)
NMR	2.995	162.790	<0.001	19.994 (12.62-31.68)
PWR	2.975	167.514	<0.001	19.592 (12.484-30.743)

**Table 7:** Multivariate Cox analysis at different variables for OS.

Variables	*β*	Wald	*P*	Hazard ratio (95% CI)
Age (years)	0.128	0.834	0.361	1.14 (0.86-1.50)
Gender	0.063	0.205	0.650	1.065 (0.81-1.395)
TNM stages	0.287	2.143	0.143	1.332 (0.907-1.96)
CA199	0.181	0.756	0.385	1.198 (0.797-1.80)
NMR	2.208	22.332	<0.001	9.095 (3.64-22.72)
PWR	2.108	20.649	<0.001	8.230 (3.32-20.43)

## Data Availability

The datasets analyzed during the current study are available from the corresponding author on reasonable request.

## References

[1] Zhang Q., Zeng L., Chen Y. (2016). Pancreatic cancer epidemiology, detection, and management. *Gastroenterology Research and Practice*.

[2] Bray F., Ferlay J., Soerjomataram I., Siegel R. L., Torre L. A., Jemal A. (2018). Global cancer statistics 2018: GLOBOCAN estimates of incidence and mortality worldwide for 36 cancers in 185 countries. *CA: a Cancer Journal for Clinicians*.

[3] Siegel R. L., Miller K. D., Jemal A. (2016). Cancer statistics, 2016. *CA: a Cancer Journal for Clinicians*.

[4] Chen W., Zheng R., Baade P. D. (2016). Cancer statistics in China, 2015. *CA: a Cancer Journal for Clinicians*.

[5] Witkiewicz A. K., McMillan E. A., Balaji U. (2015). Whole-exome sequencing of pancreatic cancer defines genetic diversity and therapeutic targets. *Nature Communications*.

[6] Wang P., Zhuang L., Zhang J. (2013). The serum miR-21 level serves as a predictor for the chemosensitivity of advanced pancreatic cancer, and miR-21 expression confers chemoresistance by targeting FasL. *Molecular Oncology*.

[7] Negoi I., Beuran M., Hostiuc S., Negoi R. I., Inoue Y. (2018). Surgical anatomy of the superior mesenteric vessels related to colon and pancreatic surgery: a systematic review and meta-analysis. *Scientific Reports*.

[8] Balkwill F., Mantovani A. (2001). Inflammation and cancer: back to Virchow?. *Lancet*.

[9] Chechlinska M., Kowalewska M., Nowak R. (2010). Systemic inflammation as a confounding factor in cancer biomarker discovery and validation. *Nature Reviews Cancer*.

[10] Diakos C. I., Charles K. A., McMillan D. C., Clarke S. J. (2014). Cancer-related inflammation and treatment effectiveness. *The Lancet Oncology*.

[11] Mantovani A., Allavena P., Sica A., Balkwill F. (2008). Cancer-related inflammation. *Nature*.

[12] Argentiero A., de Summa S., di Fonte R. (2019). Gene expression comparison between the lymph node-positive and -negative reveals a peculiar immune microenvironment signature and a theranostic role for WNT targeting in pancreatic ductal adenocarcinoma: a pilot study. *Cancers*.

[13] Gabitass R. F., Annels N. E., Stocken D. D., Pandha H. A., Middleton G. W. (2011). Elevated myeloid-derived suppressor cells in pancreatic, esophageal and gastric cancer are an independent prognostic factor and are associated with significant elevation of the Th2 cytokine interleukin-13. *Cancer Immunology, Immunotherapy*.

[14] Gabay C., Kushner I. (1999). Acute-phase proteins and other systemic responses to inflammation. *The New England Journal of Medicine*.

[15] Pichler M., Hutterer G. C., Stoeckigt C. (2013). Validation of the pre-treatment neutrophil-lymphocyte ratio as a prognostic factor in a large European cohort of renal cell carcinoma patients. *British Journal of Cancer*.

[16] Absenger G., Szkandera J., Pichler M. (2013). A derived neutrophil to lymphocyte ratio predicts clinical outcome in stage II and III colon cancer patients. *British Journal of Cancer*.

[17] Sarraf K. M., Belcher E., Raevsky E., Nicholson A. G., Goldstraw P., Lim E. (2009). Neutrophil/lymphocyte ratio and its association with survival after complete resection in non-small cell lung cancer. *The Journal of Thoracic and Cardiovascular Surgery*.

[18] Yang J. J., Hu Z. G., Shi W. X., Deng T., He S. Q., Yuan S. G. (2015). Prognostic significance of neutrophil to lymphocyte ratio in pancreatic cancer: a meta-analysis. *World Journal of Gastroenterology*.

[19] Luo G., Guo M., Liu Z. (2015). Blood neutrophil-lymphocyte ratio predicts survival in patients with advanced pancreatic cancer treated with chemotherapy. *Annals of Surgical Oncology*.

[20] Shirai Y., Shiba H., Haruki K. (2017). Preoperative platelet-to-albumin ratio predicts prognosis of patients with pancreatic ductal adenocarcinoma after pancreatic resection. *Anticancer Research*.

[21] Sakamoto T., Saito H., Amisaki M., Tokuyasu N., Honjo S., Fujiwara Y. (2019). Combined preoperative platelet-to-lymphocyte ratio and serum carbohydrate antigen 19-9 level as a prognostic factor in patients with resected pancreatic cancer. *Hepatobiliary & Pancreatic Diseases International*.

[22] Tingle S. J., Severs G. R., Goodfellow M., Moir J. A., White S. A. (2018). NARCA: a novel prognostic scoring system using neutrophil-albumin ratio and Ca19-9 to predict overall survival in palliative pancreatic cancer. *Journal of Surgical Oncology*.

[23] Clark E. J., Connor S., Taylor M. A., Madhavan K. K., Garden O. J., Parks R. W. (2007). Preoperative lymphocyte count as a prognostic factor in resected pancreatic ductal adenocarcinoma. *HPB*.

[24] Domínguez I., Crippa S., Thayer S. P. (2008). Preoperative platelet count and survival prognosis in resected pancreatic ductal adenocarcinoma. *World Journal of Surgery*.

[25] Szkandera J., Stotz M., Absenger G. (2014). Validation of C-reactive protein levels as a prognostic indicator for survival in a large cohort of pancreatic cancer patients. *British Journal of Cancer*.

[26] Grivennikov S. I., Greten F. R., Karin M. (2010). Immunity, inflammation, and cancer. *Cell*.

[27] Bausch D., Pausch T., Krauss T. (2011). Neutrophil granulocyte derived MMP-9 is a VEGF independent functional component of the angiogenic switch in pancreatic ductal adenocarcinoma. *Angiogenesis*.

[28] Russano M., Napolitano A., Ribelli G. (2020). Liquid biopsy and tumor heterogeneity in metastatic solid tumors: the potentiality of blood samples. *Journal of Experimental & Clinical Cancer Research*.

[29] Jensen H. K., Donskov F., Marcussen N., Nordsmark M., Lundbeck F., von der Maase H. (2009). Presence of intratumoral neutrophils is an independent prognostic factor in localized renal cell carcinoma. *Journal of Clinical Oncology*.

[30] He J. R., Shen G. P., Ren Z. F. (2012). Pretreatment levels of peripheral neutrophils and lymphocytes as independent prognostic factors in patients with nasopharyngeal carcinoma. *Head & Neck*.

[31] Moses K., Brandau S. (2016). Human neutrophils: their role in cancer and relation to myeloid-derived suppressor cells. *Seminars in Immunology*.

[32] Pillay J., Tak T., Kamp V. M., Koenderman L. (2013). Immune suppression by neutrophils and granulocytic myeloid-derived suppressor cells: similarities and differences. *Cellular and Molecular Life Sciences*.

[33] Nishijima T. F., Muss H. B., Shachar S. S., Tamura K., Takamatsu Y. (2015). Prognostic value of lymphocyte-to-monocyte ratio in patients with solid tumors: a systematic review and meta-analysis. *Cancer Treatment Reviews*.

[34] Hopkins A. C., Yarchoan M., Durham J. N. (2018). T cell receptor repertoire features associated with survival in immunotherapy-treated pancreatic ductal adenocarcinoma. *JCI Insight*.

[35] Shen T., Zhou L., Shen H. (2017). Prognostic value of programmed cell death protein 1 expression on CD8+ T lymphocytes in pancreatic cancer. *Scientific Reports*.

[36] Nieswandt B., Hafner M., Echtenacher B., Männel D. N. (1999). Lysis of tumor cells by natural killer cells in mice is impeded by platelets. *Cancer Research*.

[37] Klinger M. H., Jelkmann W. (2002). Role of blood platelets in infection and inflammation. *Journal of Interferon & Cytokine Research*.

[38] Ege H., Gertz M. A., Markovic S. N. (2008). Prediction of survival using absolute lymphocyte count for newly diagnosed patients with multiple myeloma: a retrospective study. *British Journal of Haematology*.

[39] Chiang S. F., Hung H. Y., Tang R. (2012). Can neutrophil-to-lymphocyte ratio predict the survival of colorectal cancer patients who have received curative surgery electively?. *International Journal of Colorectal Disease*.

[40] Aceto N., Bardia A., Miyamoto D. T. (2014). Circulating tumor cell clusters are oligoclonal precursors of breast cancer metastasis. *Cell*.

[41] Abdallah E. A., Braun A. C., Flores B. (2019). The potential clinical implications of circulating tumor cells and circulating tumor microemboli in gastric cancer. *The Oncologist*.

[42] Ben Q., An W., Wang L., Wang W., Yu L., Yuan Y. (2015). Validation of the pretreatment neutrophil-lymphocyte ratio as a predictor of overall survival in a cohort of patients with pancreatic ductal adenocarcinoma. *Pancreas*.

[43] Onoe S., Maeda A., Takayama Y. (2019). The prognostic impact of the lymphocyte-to-monocyte ratio in resected pancreatic head adenocarcinoma. *Medical Principles and Practice*.

[44] Li G. J., Xu H. W., Ji J. J., Yang F., Gao B. Q. (2016). Prognostic value of preoperative lymphocyte-to-monocyte ratio in pancreatic adenocarcinoma. *Oncotargets and Therapy*.

[45] Stotz M., Szkandera J., Stojakovic T. (2015). The lymphocyte to monocyte ratio in peripheral blood represents a novel prognostic marker in patients with pancreatic cancer. *Clinical Chemistry and Laboratory Medicine*.

[46] Smith R. A., Bosonnet L., Raraty M. (2009). Preoperative platelet-lymphocyte ratio is an independent significant prognostic marker in resected pancreatic ductal adenocarcinoma. *American Journal of Surgery*.

[47] Shirai Y., Shiba H., Sakamoto T. (2015). Preoperative platelet to lymphocyte ratio predicts outcome of patients with pancreatic ductal adenocarcinoma after pancreatic resection. *Surgery*.

[48] Hayasaki A., Isaji S., Kishiwada M. (2018). Survival analysis in patients with pancreatic ductal adenocarcinoma undergoing chemoradiotherapy followed by surgery according to the International Consensus on the 2017 Definition of Borderline Resectable Cancer. *Cancers*.

